# Plasma exosome miR-196a and miR-1246 are potential indicators of localized pancreatic cancer

**DOI:** 10.18632/oncotarget.20332

**Published:** 2017-08-18

**Authors:** Yi-Fan Xu, Bethany N. Hannafon, Yan D. Zhao, Russell G. Postier, Wei-Qun Ding

**Affiliations:** ^1^ Department of Pathology, University of Oklahoma Health Sciences Center, Oklahoma City, Oklahoma, OK 73104, USA; ^2^ Department of Biostatistics and Epidemiology, University of Oklahoma Health Sciences Center, Oklahoma City, Oklahoma, OK 73104, USA; ^3^ Department of Surgery, University of Oklahoma Health Sciences Center, Oklahoma City, Oklahoma, OK 73104, USA

**Keywords:** exosome, microRNAs, pancreatic cancer, plasma biomarkers, early detection

## Abstract

Patients with localized pancreatic cancer (stage I and stage IIA) have a much higher survival rate than those presenting at later stages, yet early detection remains a challenge to this malignancy. The aim of this study was to evaluate whether exosome miRNA signatures are indicative of localized pancreatic cancer. Exosomes were collected from the conditioned media of pancreatic cancer cell lines and plasma samples of localized pancreatic cancer patients (Stage I-IIA, n=15), and healthy subjects (n=15). Cellular and exosome miRNAs from pancreatic cancer cell lines were profiled by next-generation small RNA sequencing. Plasma exosome miRNA expression was analyzed by qRT-PCR. We found that certain miRNAs, such as miR-196a and miR-1246, are highly enriched in pancreatic cancer exosomes. Consistently, plasma exosome miR-196a and miR-1246 levels were significantly elevated in pancreatic cancer patients as compared to healthy subjects. An analysis of the cancer subtypes indicated that plasma exosome miR-196a is a better indicator of pancreatic ductal adenocarcinoma (PDAC), whereas plasma exosome miR-1246 is significantly elevated in patients with intraductal papillary mucinous neoplasms (IPMN). In contrast, there were no differences in the plasma exosome miR-196a and miR-1246 levels between patients with pancreatic neuroendocrine tumors (NET) and healthy subjects. In conclusion, we demonstrate that certain miRNA species, such as miR-196a and miR-1246, are highly enriched in pancreatic cancer exosomes and elevated in plasma exosomes of patients with localized pancreatic cancer.

## INTRODUCTION

Pancreatic cancer is the fourth-leading cause of cancer deaths in the United States [1]. The 5-year survival rate for pancreatic cancer patients was 7.8% from 2006 to 2012 [2], contrasting to a 66.9% 5-year survival rate for cancer at all sites [3]. The high mortality rate is primarily attributed to late stage diagnoses, when treatment options are often limited, and to the aggressive nature of pancreatic cancer with KRAS being a master regulator [4, 5]. Statistics have shown that localized pancreatic cancer (stage I-IIA) has a much greater 5-year survival rate (29.3%) than pancreatic cancer at later stages (11% for regionally invasive and 2.6% for distant metastasis) [2, 6]. Therefore new strategies for early detection, especially for detecting pancreatic cancer at the localized stage, are critical to improving pancreatic cancer outcomes.

There are several challenges for early detection of pancreatic cancer. First, the clinical signs and symptoms of pancreatic cancer, such as weight loss, abdominal pain, and jaundice, often manifest at a late stage and are not generally specific to pancreatic cancer [7]. Second, screening by radiological imaging of pancreatic cancer in the general population is impractical due to the high cost (*e.g.* computed tomography) or invasive procedures (*e.g.* endoscopic ultrasound or endoscopic cholangiopancreatography). Third, a sensitive and specific circulating biomarker for effective screening of pancreatic cancer is not currently available. Diagnostic evaluation often relies on the patient symptoms and appropriate imaging examination, with computed tomography (CT) being the most common initial test for identifying a pancreatic mass [8]. Cancer antigen 19-9 (CA 19-9) has been utilized for pancreatic cancer detection and the plasma level of CA 19-9 has been shown to be elevated in 75-85% of pancreatic cancer patients [9, 10]. However, circulating CA 19-9 is not specific to pancreatic cancer [11]. Furthermore it has reported that only 40% of stage I pancreatic cancer patients are CA 19-9 positive [12]. New approaches for the development of circulating biomarkers to detect localized pancreatic cancer are urgently needed in order to better manage this devastating disease.

Deregulated microRNA (miRNA) expression in pancreatic cancer cells and tissues has been reported by different groups [13–17]. Because pancreatic cancer cells display different miRNA profiles compared to normal pancreatic ductal cells, circulating miRNAs have been explored as biomarkers for pancreatic cancer [18–27]. However, a miRNA signature has not been established for clinical diagnosis or screening of pancreatic cancer. A major obstacle in the process of developing miRNAs as biomarkers for pancreatic cancer is presumably the heterogeneous nature of the circulating miRNA populations. The miRNAs isolated from the circulation are undoubtedly derived from a variety of cell types and molecular complexes. This intrinsic heterogeneity likely compromises the sensitivity and specificity of circulating miRNA detection.

Exosomes are endosome-derived membrane-bound vesicles that are secreted from different cell types and present in almost all biological fluids [28, 29]. Exosomes are 40-100 nm in size, and contain proteins, lipids and miRNAs derived from their parental cell [30–32]. Studies have shown that cancer cells secrete more exosomes than normal cells, and that cancer exosomes are released into the tumor microenvironment and circulation [33–38], suggesting that exosome contents, such as miRNAs, are potential biomarkers to improve the sensitivity and specificity of cancer detection [32, 36, 39–41]. Specific to pancreatic cancer, a recent study reported that cancer specific exosomes are detectable in patient serum that can indicate the presence of pancreatic cancer [42]. However, circulating exosome miRNAs have not been well-evaluated as biomarkers for localized pancreatic cancer largely due to the highly limited availability of the clinical specimens. To determine whether exosome miRNA signatures are indicative of localized pancreatic cancer, we characterized the miRNA content of exosomes derived from pancreatic cancer cells and analyzed candidate miRNAs in plasma exosomes from patients with localized pancreatic cancer. We report here that two highly enriched miRNAs in pancreatic cancer exosomes, miR-196a and miR-1246, are potential indicators of localized pancreatic cancer and may serve as circulating biomarkers for early detection of this malignancy.

## RESULTS

### Isolation and characterization of pancreatic cancer cell-derived exosomes

**Figure 1:**
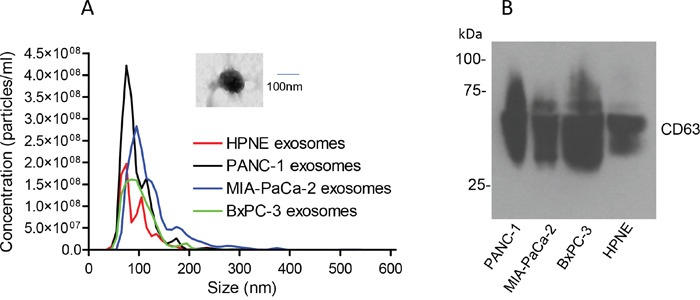
Characterization of exosomes isolated from pancreatic cancer cell lines Exosomes were isolated from the conditioned media of PANC-1, MIA-PaCa-2, BxPC-3 and hTERT-HPNE cells. **(A)** Nanoparticle tracking analysis of 50X diluted exosomes (n=3). Inset shows an exosome image observed under EM (30,000x). **(B)** Western blot of CD63 (30-75 kDa) under non-reducing conditions.

PANC-1, MIA-PaCa-2, BxPC-3 and hTERT-HPNE cells were cultured for 3 days, and whole exosomes were isolated from the conditioned medium by ultracentrifugation. The isolated exosomes were subjected to nanoparticle tracking analysis. As shown in Figure 1, the size of the exosomes from these four cell lines were comparable, ranging around 100 nm in diameter, similar to our recent reports [43, 44]. The isolated exosomes had detectable CD63, a well-established surface marker for exosomes, as evidenced by western blot analysis (Figure 1B). The PANC-1-derived exosomes were also verified by electron microscopy (EM) (Figure 1A, inset) as we recently described [44].

### miRNA profiling of PANC-1- and HPNE-derived exosomes

**Figure 2:**
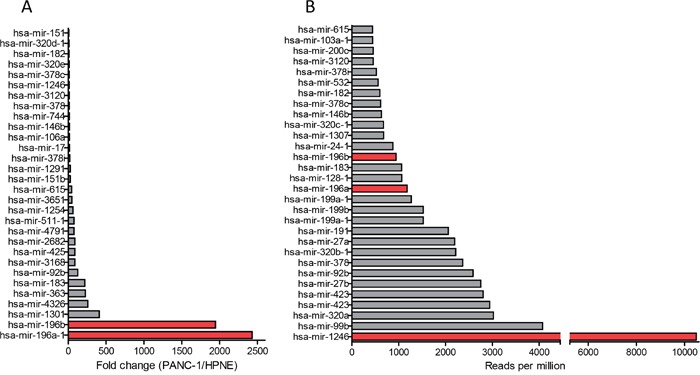
Selectively expressed and highly enriched miRNAs in pancreatic cancer exosomes Cellular and exosome RNA was isolated from hTERT-HPNE and PANC-1 cells. microRNA expression was analyzed using Small RNA next generation sequencing (n=3). (**A**) Top 30 miRNAs highly expressed in PANC-1 exosomes compared to hTERT-HPNE exosomes (fold of changes). (**B**) Top 30 miRNAs highly abundant in PANC-1 exosomes (reads per million).

**Figure 3:**
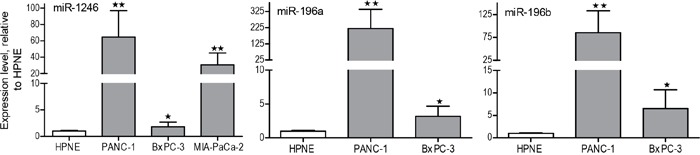
qRT-PCR analysis of miR-1246, miR-196a and miR-196b expression in PANC-1, MIA-PaCa-2, and BxPC-3 exosomes versus hTERT-HPNE exosomes Total RNA was extracted from the exosomes and miR-196a, miR196b and miR-1246 expression was measured by qRT-PCR. Fold-change in expression is shown for miR-1246, miR-196a and miR-196b relative to their levels in hTERT-HPNE exosomes and normalized to the spike-in cel-miR-54 control (n=3, means ± SEM). *p<0.05; **p<0.01; Student's t-test.

Total RNA was isolated from PANC-1- and hTERT-HPNE-derived exosomes and a small RNA library was generated. The cDNA library was sequenced by Illumina sequencers, and the sequences were analyzed against the miRBase database and quantified as reads per million for each individual miRNA species. There were around 150 miRNAs detected in exosomes derived from both cell lines (Supplementary Table 1). By comparing miRNA levels in PANC-1 and hTERT-HPNE exosomes, we identified the top 30 miRNAs that are highly expressed in PANC-1 exosomes compared to hTERT-HPNE exosomes (Figure 2A). We also identified top 30 highly abundant miRNAs within PANC-1 exosomes (Figure 2B). We selected miR-196a, miR-196b and miR-1246 as candidate exosome miRNAs for biomarker development, due to their selective expression and high abundance in PANC-1 exosomes (Table 1). Expression of exosome miR-196a, miR-196b, and miR-1246 was verified by qRT-PCR analysis using exosomes derived from hTERT-HPNE, PANC-1, MIA-PaCa-2, and BxPC-3 cells. Significantly higher levels of these three miRNAs were evident in exosomes derived from cancer cells as compared to exosomes derived from hTERT-HPNE cells (Figure 3), consistent with the RNA sequencing results. We therefore focused on exosome miR-196a, miR-196b and miR-1246 in the following experiments.

**Table 1:** Expression of miR-1246, miR-196a and miR-196b in PANC-1 and hTERT-HPNE exosomes (reads per million)

miRNA	Group MeanHPNE Exo	Group MeanPANC-1 Exo	PANC-1 Exo/HPNE Exo
miR-1246	815.942	10481.01	12.58
miR-196a	0.48568	1179.84	2429.25
miR-196b	0.48568	944.4833	1944.66

### Plasma exosome miR-196a and miR-1246 are indicative of localized pancreatic cancer

**Figure 4:**
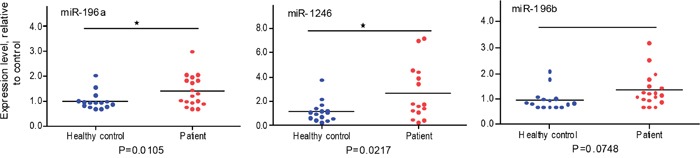
qRT-PCR analysis of plasma exosome miRNA expression in normal and pancreatic cancer patients Plasma samples were collected from healthy subjects without a history of cancer (n=15) and patients with localized pancreatic cancer (n=15; see Table 2). The Exoquick reagent was used to isolate exosomes from plasma samples. Total RNA was extracted from the plasma exosomes and miR-196a, miR196b and miR-1246 expression was measured by qRT-PCR. Cel-miR-54 was spiked-in to serve as experimental control. Data are expressed as relative expression levels. *p<0.05, Student's t-test.

**Figure 5:**
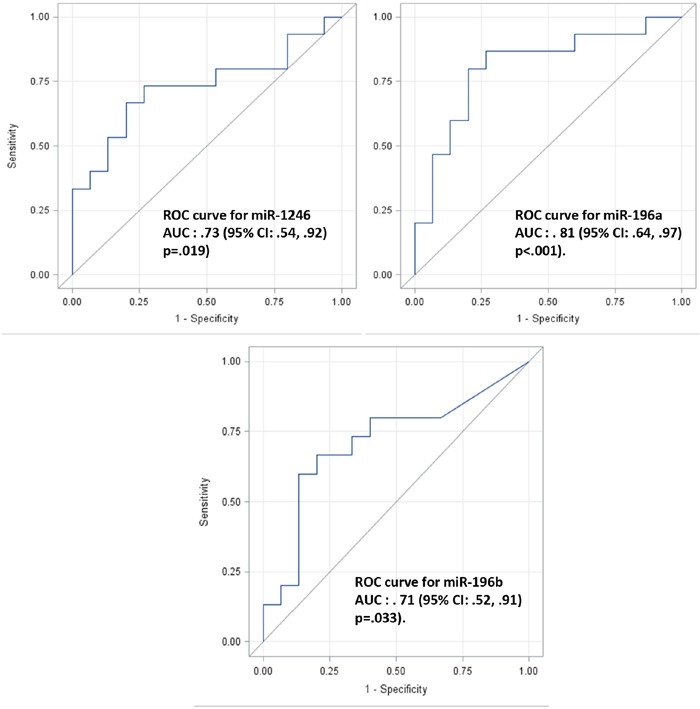
ROC analysis of microRNA expression in normal and pancreatic cancer patient plasma exosomes Receiver Operator Characteristic (ROC) curves were constructed using each microRNA expression value for miR-1246 (top left), miR-196a (top right) and miR-196b (low) respectively. The average of triplicate expression values was computed for individual patient sample and microRNA. The Area Under the Curve (AUC) with 95% CI were computed and shown for each ROC curve. The Wilcoxon-Mann-Whitney test was used to test the null hypothesis that the AUC is equal to .5 (i.e., no predictive power) and the P values for each test were shown.

To determine whether plasma exosome miRNAs are indicative of localized pancreatic cancer, 15 plasma samples from patents with localized pancreatic cancer (stage I or stage IIA; see Table 2 for patient information) and matched plasma samples from healthy controls were collected. Plasma exosomes were isolated and total RNA was extracted from the isolated plasma exosomes as we recently reported [44]. The expression levels of plasma exosome miR-1246, miR-196a and miR-196b were analyzed by qRT-PCR. As shown in Figure 4, the expression of plasma exosome miR-196a (p=0.0105) and miR-1246 (p = 0.0217) was significantly higher in the group of patients with localized pancreatic cancer as compared to the control population. No such difference was detected for miR-196b. There was no significant difference in plasma exosome miR-1246 and miR-196a expression levels between stage I and stage IIA patients and no correlation between tumor size and the plasma exosome miRNA expression levels (data not shown). Receiver operating characteristic curves (ROC) were constructed for miR-196a, miR-196b and miR-1246 to compare the diagnostic value of their plasma exosome levels in predicting stage I or stage IIA pancreatic cancer (Figure 5). The area under curve (AUC) for miR-196a was 0.81 (95 % CI 0.64, 0.97; p<0.001) and for miR-1246 was 0.73 (95 % CI 0.54, 0.92; p = 0.019), indicating their fair predictive power. The AUC for miR-196b was 0.71 (95 % CI 0.52, 0.91; p=0.033). These data show that plasma exosome miRNAs, especially miR-196a and miR-1246, are indicative of localized pancreatic cancer.

**Table 2:** Clinicopathological features of pancreatic cancer patients included in the study

Plasma Sample	Age	Gender	Diagnosis	Tumor size	Stage^a^
PL1	49	male	PDAC	2.5×2.8×3 cm	Stage IIA
PL2	85	female	PDAC	1.8×0.7×0.4 cm	Stage IIA
PL3	34	female	NET	1.4×1.2×1.8 cm	Stage I
PL4	74	female	NET	1.6×1.5×1.8 cm	Stage I
PL5	74	male	IPMN	1.4×0.5×1.6cm	Stage I
PL6	70	male	PDAC	0.6×0.4×1 cm	Stage IIA
PL7	65	female	NET	5.8×5.1×7.2 cm	Stage IIA
PL8	47	male	NET	2.5×1.7×2.7 cm	Stage IIA
PL9	84	male	PDAC	2.5×4.5 cm	Stage IIA
PL10	64	female	PDAC	0.7×0.5×0.8 cm	Stage I
PL11	60	female	PDAC	NA	Stage I
PL12	73	male	IPMN	2×1.5×1.8 cm	Stage I
PL13	67	male	IPMN	5×5×1 cm	Stage I
PL14	70	male	PDAC	1.5×1.0×2.0 cm	Stage IIA
PL15	83	female	IPMN	9×2.5×3.5 cm	Stage IIA
Mean Age	66.66				

^a^Pathologic TNM tumor staging, (American Joint Committee on Cancer Care 7^th^ Edition).

### Plasma exosome miR-196a and miR-1246 expression in subtypes of localized pancreatic cancer

**Figure 6:**
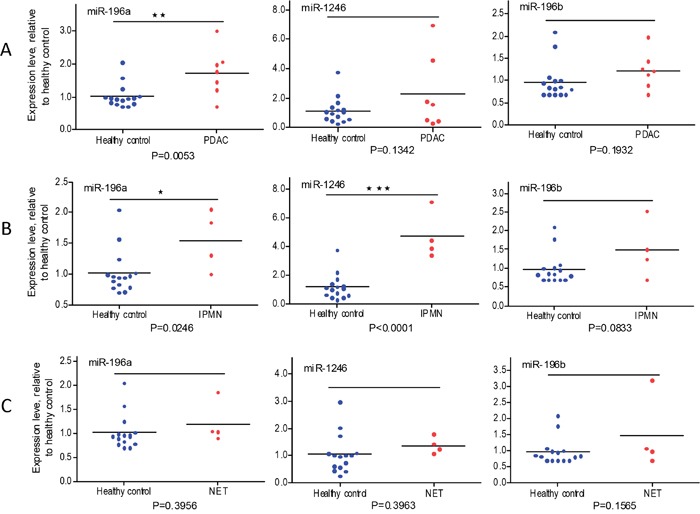
qRT-PCR analysis of plasma exosome miRNA expression in PADC, IPMN and NET patients relative to healthy subjects Expression of miR-1246, miR-196a and miR-196b was analyzed using plasma exosomes from PDAC patients (**A**, n=7), IPMN patients (**B**, n=4), NET patients (**C**, n=4), and healthy subjects (n=15). Cel-miR-54 was spiked-in to serve as experimental control. Data are expressed as relative expression levels. *p<0.05; **p<0.01; ***p<0.001; Student's t-test.

Different histologic types of pancreatic cancer have different clinical outcomes [45]. The 15 patients with localized pancreatic cancer included 7 pancreatic ductal adenocarcinomas (PDAC), 4 intraductal papillary mucinous neoplasms (IPMN), and 4 well differentiated neuroendocrine tumors (NET). Figure 6 shows that plasma exosome miR-196a is a better indicator for PDAC (Figure 6A, P=0.0053) and that plasma exosome miR-1246 levels are significantly elevated in patients with IPMN (Figure 6B, P<0.0001). Plasma exosome miR-196a levels were also significantly higher in IPMN patients (P=0.0246). There was no significant difference in plasma exosome miR-196a and miR-1246 levels in patients with NET as compared to healthy controls (Figure 6C). Similar levels of plasma exosome miR-196b were observed in all comparisons.

### Immunoaffinity isolation of plasma exosomes using a *GPC1 antibody* does not improve the diagnostic value of exosome miR-1246 for pancreatic adenocarcinoma

**Figure 7:**
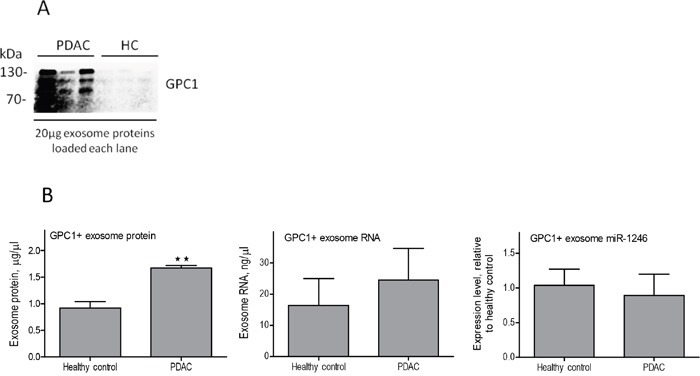
miR-1246 expression in GPC1 positive plasma exosomes Total plasma exosomes from three patients with localized pancreatic cancer and three healthy controls were subjected to immunoaffinity isolation using the GPC1 antibody complexed magnetic beads. Exosome protein and RNA were extracted from the antibody-beads complexes using TRIzol reagent. **(A)** Western blot analysis of the immunoaffinity isolated exosomes from patients (PDAC) and healthy controls (HC), using an antibody against GPC1 (equal amount of exosome protein was loaded in each lane). Note that GPC1 was detected at significantly higher levels in PDAC GPC1 positive exosomes (bands range from 70-130 kDa). **(B)** Protein concentrations in GPC1 positive plasma exosomes (left), **p<0.01, Student's t-test; RNA concentrations in GPC1 positive plasma exosomes (center), and miR-1246 expression in GPC1 positive plasma exosomes (right, n=3, means ± SEM).

A recent report demonstrated that GPC1 positive plasma exosomes are indicative of pancreatic adenocarcinoma in a large patient cohort study [42]. To explore whether miRNAs in the GPC1 positive exosomes are better indicators of localized pancreatic adenocarcinoma, we utilized GPC1 antibody-conjugated beads to isolate GPC1 positive exosomes from the total plasma exosome populations. The miR-1246 expression in GPC1 positive exosomes from 3 patients with localized pancreatic adenocarcinoma and 3 healthy donors were compared (Figure 7). miR-1246 was analyzed due to its high abundance in pancreatic cancer exosomes (Table 1) and would therefore be detectable in the limited amount of RNA isolated from the immunoaffinity isolated exosomes. There were more GPC1 positive exosomes in the patient plasma than in the control population as suggested by western blot for GPC1 after immunoaffinity isolation (Figure 7A). This was further indicated by the quantity of GPC1 positive exosome protein (Figure 7B left) in the patient plasma versus normal plasma. The GPC1 positive exosome RNA concentration was also slightly higher in patient plasma than in normal plasma even though statistical significance was not evident (Figure 7B center). However, plasma exosome miR-1246 expression levels in patients with localized pancreatic adenocarcinoma were not increased after GPC1 immunoaffinity isolation (Figure 7B right). The same is true when cell line-derived exosomes were analyzed using GPC1 immunoaffinity isolation (data not shown), suggesting that GPC1 positive exosomes do not provide an advantage for detection of localized pancreatic cancer in plasma exosome miRNA. Further verification of this conclusion using a larger set of plasma exosome samples from localized pancreatic cancer patients is warranted.

## DISCUSSION

The most interesting finding from the present study is that miR-196a and miR-1246 are highly enriched in pancreatic cancer exosomes, and that these two miRNAs are detected at significantly higher levels in plasma exosome samples from patients with localized pancreatic cancer as compared to those from matched healthy controls. These results indicate that plasma exosome miRNAs, such as miR-196a and miR-1246, are potential biomarkers for early detection of pancreatic cancer. Subtype analysis further indicated that plasma exosome miR-196a is a better indicator for localized PDAC, and plasma exosome miR-1246 is an excellent indicator for localized IPMN. Interestingly, both plasma exosome miR-196a and miR-1246 were expressed at similar levels among patients with NET and the healthy controls. Correlation of histological subtypes and miRNA expression has been described in various malignancies, including lung cancer [46], gastric cancer [47], malignant mesothelioma [48], melanoma [49], cholangiocarcinoma [50], and periampullary carcinoma [51].

miR-1246 contained in microvesicles of colon cancer cells was recently found to promote tumor angiogenesis via targeting the promyelocytic leukemia protein and the Smad signaling pathway in endothelial cells [52]. In hepatocellular carcinoma, miR-1246 was reported to enhance cell migration and invasion through reducing CADM1 expression [53], and increase cancer stemness via targeting Octamer 4 [54]. The expression levels of miR-1246 were reported to be associated with CCNG2-mediated chemoresistance and stemness in pancreatic cancer cells [55]. miR-1246 was also found to promote tumor progression in cervical [56, 57] and lung cancer [58–61]. A recent study indicated that circulating exosome miR-1246 levels are elevated in pancreatic cancer patients [62]. Similar to miR-1246, miR-196a seems to be involved in tumorigenesis and tumor progression in different types of cancer [63]. Recent reports suggested that miR-196a promotes pancreatic cancer progression by targeting the NF-κB inhibitor α [64] and high expression of miR-196a may predict poor outcomes of patients with pancreatic cancer [65]. In addition, studies have shown that circulating miR-196a, in combination with other miRNAs, may serve as biomarkers for pancreatic cancer [65, 66]. These previous reports are consistent with our current findings, suggesting that both miR-1246 and miR-196a are likely oncomiRs and may serve as diagnostic or prognostic markers for pancreatic cancer.

An important feature of this study is that we focused on plasma exosome miRNAs in patients with localized pancreatic cancer, which is at the early stage when local lymph nodes are not involved and there are no symptoms or signs manifested [2]. These patients, if treated, have a significantly higher 5 year survival rate [2]. Serum or plasma miRNAs have been explored for their potential as biomarkers for pancreatic cancer [18–27]. Recent studies have shown that exosome miRNAs are potential indicators for pancreatic cancer [41, 67]. Serum miR-1246 levels were also demonstrated to be significantly higher in pancreatic cancer patients [62]. However, most of these studies have included a general population of pancreatic cancer patients of mixed stages. To our knowledge, there have been no reports focusing exclusively on the potential of plasma or serum exosome miRNAs as biomarkers for localized pancreatic cancer. We have demonstrated in this study, in a pilot cohort of 15 patients with localized pancreatic cancer, that plasma exosome miR-196a and miR-1246 levels are significantly higher in early stage pancreatic cancer as compared to matched healthy controls. When this group of patients was divided into histologic subtypes, miR-196a was found to be a better and significant indicator of PDAC, and miR-1246 an excellent indicator of IPMN. These observations are generally in line with previous reports showing that serum levels of miR-1246 is elevated in pancreatic cancer patients [62], and that miR-196a is deregulated in pancreatic cancer tissues, including those at early stages [66]. The findings from this pilot study signify new plasma exosome miRNA signatures that are potential biomarkers for early detection of pancreatic cancer. Given the relative small patient sample size of this pilot study, as limited by the availability of localized pancreatic cancer specimens, further evaluation of these two plasma exosome miRNAs in a larger cohort is required to establish these new biomarkers for detection of localized pancreatic cancer.

Another feature of the current study, which is different from many other biomarker studies, is that we began the investigation with small RNA sequencing to characterize pancreatic cancer exosome miRNA signatures using human pancreatic cell line-derived exosomes. We have recently applied and validated this strategy for biomarker discovery in breast cancer [44]. The RNA sequencing results provided us with an indication as to which exosome miRNAs we might focus on for biomarker development. Two criteria were used to select candidate exosome miRNAs for clinical study: first, these miRNAs are selectively encapsulated in pancreatic cancer exosomes versus normal pancreatic ductal epithelial cell exosomes; and second, these miRNAs are highly abundant in pancreatic cancer exosomes. These criteria are based on the consideration that at the localized pancreatic cancer stages (stage I-IIA), there would likely have fewer cancer exosomes available in the circulation as the tumor is still localized to the pancreas without lymph node involvement and distal metastasis. We selected miR-1246, miR-196a and miR-196b for further evaluation using pancreatic cancer patient plasmas. Interestingly, while plasma exosome miR-196b expression was similar in pancreatic cancer patients and the healthy controls, which is inconsistent with cell line studies, plasma exosome miR-196a and miR-1246 expression was significantly higher in pancreatic cancer patients than in the matched healthy controls. These results indicate that starting with a characterization of human pancreatic cancer cell line-derived exosomes seems to be a valid approach for plasma exosome biomarker discovery; but one needs to be cautious as the results from cell lines may not always be applicable to patient plasma exosomes.

The ROC curve analysis for our patient study indicates that while the difference of plasma exosome miR-196a and miR-1246 expression between pancreatic cancer patients and the controls is statistically significant, the sensitivity and specificity of these plasma exosome miRNAs as indictors for localized pancreatic cancer may be further improved (see AUC for each ROC curve in Figure 5). In this context, we attempted to evaluate pancreatic cancer exosomes from whole plasma exosomes using an immunoaffinity isolation method, in order to obtain more specific and sensitive analysis of plasma exosome miRNA expression. As GPC1 was recently reported as a pancreatic adenocarcinoma exosome surface marker [42], we performed immunoaffinity isolation from whole plasma exosomes using the reported GPC1 antibody. Whereas immunoaffinity isolation with the GPC1 antibody captured more exosomes from pancreatic adenocarcinoma patient plasma samples, miR-1246 expression in GPC1 positive exosomes is not elevated in patients with localized PDAC compared to the matched controls. This unexpected result suggests that immunoaffinity isolation using GPC1 antibodies for plasma exosome miRNA analysis may not be an ideal approach to capture differentially expressed exosome miRNAs in patients with localized PDAC. New strategies are needed to furthering this avenue of investigation.

In conclusion, we have demonstrated that certain miRNAs, such as miR-196a and miR-1246, are highly and selectively enriched in pancreatic cancer cell-derived exosomes and that the plasma exosome levels of these two miRNAs are significantly elevated in patients with localized pancreatic cancer. Plasma exosome miR-196a and miR-1246 may serve as biomarkers for different subtypes of localized pancreatic cancer, with miR-196a for PDAC and miR-1246 for IPMN. As early diagnosis remains the key to improving pancreatic cancer outcomes, further studies are warranted to firmly establish plasma exosome miRNA signatures indicative of localized pancreatic cancer.

## MATERIALS AND METHODS

### Cell culture

The human pancreatic epithelial cell line hTERT-HPNE, and pancreatic cancer cell lines PANC-1, MIA-PaCa-2 and BxPC-3 were obtained from the American Type Culture Collection (ATCC, Manassas, VA, USA). Cells were cultured according to ATCC's instructions with the exception of exosome-depleted fetal bovine serum (FBS) and horse serum being used wherever appropriate. Exosome-depleted FBS and horse serum were prepared by pelleting the serum exosomes by ultracentrifugation at 100,000 × g for 2 h at 4°C, and the resulting supernatant was filtered through a 0.2-μm pore filter. Cells were routinely maintained in a humidified chamber at 37°C and 5% CO2.

### Patient plasma samples

Plasma samples (n=15) were collected from donors with no history of pancreatic cancer (mean age = 48, 11 females and 4 males) from the Oklahoma Blood Institute, Oklahoma City, OK, USA under an approved IRB protocol. Localized pancreatic cancer plasma samples (n=15, Table 2) were collected from patients who were seen at the Stephenson Cancer Center at the University of Oklahoma Health Science Center (OUHSC), and underwent primary tumor biopsy or resection. The study was approved by the OUHSC IRB (#5535) and written informed consent was obtained from all participants. Blood was collected before biopsy or resection as described [44]. Plasma was separated by centrifugation and stored at -80 °C. Clinical stages were classified using the tumor, node, metastasis (TNM) system (AJCC 7th edition). Patient information including age, tumor size, clinical stages and histological grades were obtained from the clinical and pathological records.

### Exosome isolation

Exosomes were isolated from the culture medium utilizing a combination of centrifugation, ultracentrifugation, and filtration as we previously described [43, 44]. Plasma exosomes were isolated using the Exoquick reagent (System Biosciences, Mountain View, CA, USA) following the manufacturer's protocol (500μl plasma per sample).

### Western blot analysis

Total exosome protein was prepared by resuspending the exosomes in RIPA Buffer (50mM Tris-HCl pH 7.4, 150mM NaCl, 0.5% sodium deoxycholate, 1% NP-40, and 0.1% sodium dodecyl sulfate) containing 1 mM phenlymethylsulfonyl fluoride, 5μg/ml leupeptin, 2μg/ml aprotinin, and 1μg/ml pepstatin A [44]. Approximately 30–40μg of protein from each sample was separated under non-reducing conditions (for CD63) or reducing conditions (for glypican-1 (GPC1)) on a 10% SDS-PAGE gel, transferred to a polyvinylidene fluoride membrane, blotted with a primary antibody against CD63 (sc-5275, Santa Cruz Biotechnology, Santa Cruz, CA, USA), GPC1 (PA-24972, Thermo Scientific, Waltham, MA USA), and a secondary IgG-HRP (Santa Cruz Bio Technology Inc., CA, USA).

### Electron microscopy

Exosomes were suspended in 1 × PBS and fixed with 2% paraformaldehyde. The fixed sample was absorbed onto formvar-coated copper grids for 20 min in a dry environment and fixed in 1% glutaraldehyde for 5 min. After being rinsed in distilled water, samples were stained with uranyl oxalate for 5 min followed by methyl cellulose uranyl acetate for 10 min on ice. Excess liquid was wicked off the grid, and grids were stored at room temperature until imaging. Imaging was performed using a Hitachi H7600 electron microscope.

### Nanoparticle tracking analysis

Isolated exosomes were diluted in PBS and analyzed using the Nanosight NS300 System (Malvern Instruments, UK) which is equipped with a blue laser (405 nm) [44]. Nanoparticles illuminated by the laser were captured for 60 seconds. The Nanosight Tracking Analysis software was used to provide particle concentrations and size distribution profiles.

### RNA extraction

Total RNA was extracted from exosome pellets using the TRIzol reagent (Invitrogen/Life Technologies, Carlsbad, California) following the manufacturer's protocol [44]. RNA concentration was quantitated using the NanoDrop ND-100 Spectrophotometer (NanoDrop Technologies, Wilmington, DE, USA).

### Small RNA library preparation and next generation sequencing

Small RNA libraries were constructed using the New England Biolabs (NEB) NEBNext Multiplex Small RNA Library Prep Set for Illumina sequencers and the NEB standard protocol, as we recently described [44]. Each library was indexed to multiplex four samples per sequencing run on the Illumina MiSeq platform using MiSeq 50 cycle Reagent Kits v2. A minimum of 17 million 50-bp sequencing reads were collected from each sample and data were analyzed using the Genesifter software (formerly Geospiza, PerkinElmer, Santa Clara, CA, USA). Raw data for each sample were aligned to the most recent miRBASE database (miRBase.org [68]). Pairwise comparisons of the alignment results for identification of miRNAs that are differentially expressed at a significant level were conducted using the Genesifter software.

### Quantitative real-time reverse transcription PCR

For miRNA expression analysis, complementary DNA from 80 ng of total RNA was synthesized using the qScript miRNA cDNA Synthesis Kit (Quanta BioSciences, Inc., Beverly, MA, USA). A cDNA aliquot, equivalent of 4 ng of the original RNA, was mixed with Perfecta SYBR Green SuperMix, Universal PCR Primer and specific forward primers for miR-1246 (5’-GCGCGATGGATTTTTGGAGCAG-3’), miR-196a (5’-GCGTAGGTAGTTTCCTGTTGTTGGG-3’), or miR-196b (5’-GCGTAGGTAGTTTCCTGTTGTTGGG-3’) in 20 μl qPCR reactions. A synthetic *Caenorhabditis elegans* miR-54 (cel-miR-54) RNA oligonucleotide (Integrated DNA Technologies, Coralville, IA, USA) was spiked-into RNA samples as a control [69] (5’-UACCCGUAAUCUUCAUAAUCCGAG-3’). PCR reactions were run on a Bio-Rad CFX 96 Real-Time PCR (Bio-Rad, Hercules, CA, USA) instrument under following conditions: 95 °C for 2 min, 40 cycles of 95 °C for 5 s, 60 °C for 15 s and 70°C for 15 s. Changes in gene expression were calculated using the ΔΔ*C_T_* method as follows: Δ*C_T_* = *C_T_*(target miRNA) − *C_T_* (cel-miR-54); ΔΔ*C_T_ =* Δ*C_T_* (cancer exosome miRNA)- Δ*C_T_* (normal exosome miRNA); the fold change = 2^−ΔΔ*CT*^.

### Immunoaffinity magnetic bead-based exosome isolation

Protein G Dynabeads were purchased from Thermo Fisher (Waltham, MA, USA). The process of preparing GPC1 antibody (PA-24972, Thermo Scientific, Waltham, MA USA) coated beads and immunoaffinity capture of the exosomes were described in our recent report [44]. Proteins and RNA was extracted from the exosome-bound beads using the TRIzol reagent.

### Statistical analysis

Statistical analyses were completed using GraphPad Prism software (GraphPad Software, Inc. La Jolla, CA, USA) and SAS software (SAS institute, Cary, North Carolina, USA). Student's t test was used to determine significant differences among control and experimental groups. Receiver operating characteristic (ROC) curves were constructed using each miRNA expression value. The area under the curve (AUC) with 95% CI was calculated for each ROC curve. The Wilcoxon-Mann-Whitney test was used to test the null hypothesis that the AUC is equal to 0.5 (i.e., no predictive power).

## SUPPLEMENTARY MATERIALS AND TABLES



## Abbreviations

CA 19-9: Cancer antigen 19-9
AUC: Area under the curve
FBS: fetal bovine serum
GPC1: glypican-1
miRNA: microRNA
IPMN: intraductal papillary mucinous neoplasms
NET: neuroendocrine tumors
NEB: New England Biolabs
PDAC: polyvinylidene fluoride
PVDF: pancreatic ductal adenocarcinomas
qRT-PCR: Quantitative Real-Time PCR
ROC: Receiver operating characteristic
