# Supplementary material for: Plasma exosome miR-196a and miR-1246 are potential indicators of localized pancreatic cancer
Source: Oncotarget. 2017 Aug 18;8(44):77028–40. doi: 10.18632/oncotarget.20332 (PMC5652761; doi:10.18632/oncotarget.20332)
Supplement: Supplementary file 1 [file oncotarget-08-77028-s001.pdf]

Plasma exosome miR-196a and miR-1246 are potential indicators of localized pancreatic cancer

SUPPLEMENTARY MATERIALS

Supplementary Table 1: Small RNA sequencing results of HPNE- and PANC-1-derived exosomes

See Supplementary File 1
